# Functional Characterization of *AfBBX* from *Amorpha fruticosa* in Enhancing Osmotic and Salt–Alkali Tolerance in Transgenic Tobacco

**DOI:** 10.3390/ijms27114902

**Published:** 2026-05-28

**Authors:** Mengwen Wei, Hanyu Zhang, Yifan Wang, Jianan Guo, Qingjie Guan

**Affiliations:** 1 Aulin College, Northeast Forestry University, Harbin 150040, China; 2023224802@nefu.edu.cn (M.W.); 2023224810@nefu.edu.cn (H.Z.); 2023214756@nefu.edu.cn (Y.W.); 2 Key Laboratory of Saline–alkali Vegetation Ecology Restoration, Ministry of Education, College of Life Sciences, Northeast Forestry University, Harbin 150040, China; gjn00@nefu.edu.cn

**Keywords:** *Amorpha fruticosa*, *AfBBX*, BBX protein, abiotic stress, zinc finger protein, tobacco, genetic transformation

## Abstract

Drought and soil salinization severely limit the productivity of global agriculture and forestry, highlighting the urgency of identifying stress-resistant genes for molecular breeding. B-box (BBX) proteins constitute a class of zinc finger transcription factors that play significant roles in plant abiotic stress responses. *Amorpha fruticosa* (*A. fruticosa*) is a perennial woody plant with exceptional adaptability to harsh environments, serving as a valuable resource for mining stress-resistant genes. In this study, the *AfBBX* gene was cloned from *A. fruticosa*, and its function in stress tolerance was systematically analyzed. Bioinformatics analysis confirmed that *AfBBX* contains a conserved ZnF-BBOX domain and shares functional conservation with the BBX protein family. Quantitative real-time polymerase chain reaction (qRT-PCR) revealed tissue-specific expression of *AfBBX*, with the highest expression in stems and the lowest in young leaves. Furthermore, *AfBBX* expression was dynamically regulated in roots and leaves of *A. fruticosa* under treatments of 5 μM ABA (drought mimic), H_2_O_2_ (oxidative stress), 10% PEG600 (osmotic stress), and NaHCO_3_ (alkaline stress). Transgenic tobacco lines overexpressing *AfBBX* showed enhanced tolerance to osmotic and salt–alkali stresses at both germination and seedling stages. Meanwhile, compared to wild-type (WT) tobacco, transgenic lines exhibited higher germination rates, longer root lengths, and greater fresh weights under stress conditions. Under natural drought and salt–alkali stresses, transgenic tobacco maintained higher chlorophyll fluorescence intensity (Fv/Fm values), elevated activities of antioxidant enzymes [superoxide dismutase (SOD)], and reduced malondialdehyde (MDA) content. In conclusion, *AfBBX* enhances stress tolerance by mitigating photosystem damage, increasing reactive oxygen species (ROS) scavenging capacity, and reducing membrane lipid peroxidation. The findings from this study provide novel insights into the molecular mechanism underlying *AfBBX*-mediated stress resistance and offer valuable genetic resources for breeding drought- and salt-tolerant crops and forest trees.

## 1. Introduction

Forests are vital components of terrestrial ecosystems with outstanding carbon sequestration capacities, storing approximately 80% of aboveground and 40% of belowground terrestrial carbon [1]. However, owing to the current changing climate that induces soil drought and salinization, plants, as sessile organisms, are constantly exposed to multiple environmental stresses, which produce detrimental effects on their growth and development [2,3]. It is projected that the extent of arable and forested land will be further reduced by the ongoing aridification trends and secondary salinization, thereby compromising global food security and ecological resilience [4]. In this context, there is an urgent need to prioritize the elucidation of the molecular genetic basis of plant stress adaptation and the development of resilient germplasm through molecular breeding.

To cope with abiotic stresses, woody plants have evolved intricate regulatory networks, among which transcription factors (TFs) serve as central hubs that perceive stress signals and orchestrate downstream gene expression [5,6]. Among the diverse TF families, B-box (BBX) proteins, characterized by the presence of one or two conserved zinc-finger BBX (ZnF-BBOX) domains, have emerged as pivotal regulators integrating light signaling, photomorphogenesis, and abiotic stress responses [7,8]. Regarding their well-established mechanistic involvement in stress adaptation, in *Arabidopsis thaliana*, *AtBBX24*/SALT TOLERANCE enhances salinity tolerance by modulating reactive oxygen species (ROS) homeostasis and flavonoid biosynthesis [9,10]. Similarly, *OsBBX17* in rice (*Oryza sativa*) positively regulates the abscisic acid (ABA) signaling pathway to confer drought resistance [11]. Furthermore, there is species-specific functional diversity: *MdBBX37* improves cold tolerance in apple (*Malus domestica*) [12], whereas *SlBBX18* acts as a negative regulator of drought resistance in tomato (*Solanum lycopersicum*) [13]. In forest trees, *PfBBX13* and *PfBBX14* from *Paulownia* [14], as well as *GkBBX25* from *Ginkgo biloba* [15], enhance drought and salt tolerance, respectively, highlighting the potential of *BBX* genes in engineering multi-stress resilience in perennial woody species. Recent advances have facilitated the genome-scale identification of *BBX* family members across various plant species, including studies that combine expression profiling with transient transformation assays to characterize their stress-responsive functions [16].

To discover novel and robust genetic resources, exploring stress-adapted wild species, termed “extremophytes,” represents a powerful strategy [17]. *Amorpha fruticosa* L. (*A. fruticosa*) is a perennial leguminous shrub that has a wide distribution across arid, semi-arid, and saline–alkaline regions of Northeast Asia. This type of plant exhibits remarkable tolerance to concurrent drought, salinity, and alkaline stress, making it an ideal system for probing natural adaptation mechanisms [18,19]. As identified by existing functional studies, several key stress-responsive genes have been characterized from this species. For instance, C2H2-type zinc finger proteins have been shown to enhance drought tolerance via the auxin signaling pathway, as demonstrated by the overexpression of TaZAT8-5B in transgenic Arabidopsis [20]. Similarly, NAC transcription factors, such as LpNAC5 from Lilium pumilum, positively regulate drought, salt, and alkaline tolerance when heterologously expressed in tobacco [21]. Moreover, WRKY transcription factors have been extensively documented to play essential roles in drought stress responses, acting as central regulators in plant abiotic stress signaling networks [22]. However, despite the recognized roles of BBX proteins in abiotic stress responses and the acknowledged value of *A. fruticosa* as a genetic reservoir, the functions and regulatory mechanisms of BBX family members in this highly resilient species remain entirely unexplored. Therefore, we hypothesized that the *AfBBX* gene from *A. fruticosa* functions as a positive regulator of drought and saline–alkali stress tolerance, and that its heterologous expression in tobacco would enhance stress resistance by improving photosynthetic efficiency, increasing antioxidant enzyme activity, and alleviating oxidative damage.

Given the impending requirement for developing stress-resistant woody plants to achieve ecological restoration and sustainable forestry, the present study focused on *A. fruticosa*, and achieved successful cloning and identification of a novel *BBX* gene, designated *AfBBX*, coupled with a systematic investigation of its role within the abiotic stress response network. This study first analyzed the sequence and structural features of *AfBBX*, along with its tissue-specific expression patterns, with the aim of elucidating the molecular function of this previously uncharacterized BBX gene in a stress-tolerant woody plant. *AfBBX* was then investigated as a putative positive regulator of abiotic stress tolerance. Furthermore, its heterologous expression in tobacco was implemented to assess its functional roles, focusing on the protection of the photosynthetic apparatus, enhancement of ROS scavenging, and alleviation of membrane lipid peroxidation. This study comprehensively evaluated the role of *AfBBX* in conferring drought and saline–alkali stress tolerance, demonstrating its potential as a valuable genetic resource for molecular breeding programs to facilitate the future development of drought-resistant and salt-tolerant forest trees.

Beyond providing an initial functional characterization of *AfBBX* in *A. fruticosa*, this work also offers a promising genetic resource for molecular breeding aimed at enhancing drought and salt resilience in crops and forest trees. Furthermore, it may deepen our understanding of convergent and divergent evolutionary strategies employed by stress-responsive TFs in herbaceous model plants versus wild perennial species. Ultimately, these findings may provide new insights for designing multi-layered genetic improvement strategies to address compounded climate stressors.

## 2. Results

### 2.1. Cloning and Bioinformatics Analysis of the AfBBX

**Figure 1:**
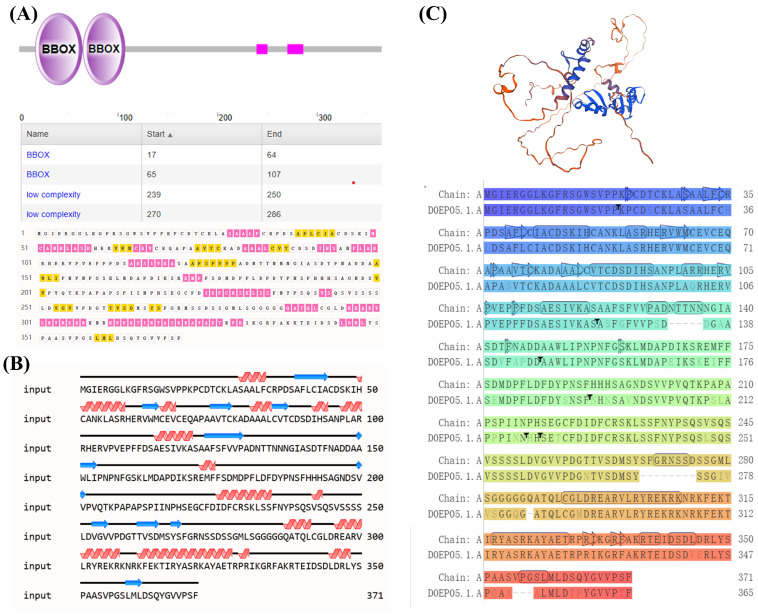
Bioinformatics analysis of *AfBBX*. (**A**) Predicted primary structure of the *AfBBX* protein by SMART, showing two ZnF-BBOX domains (red boxes). Purple lines represent low-complexity domains. In the sequence diagram at the bottom, the ZnF-BBX domain is highlighted in yellow and the low-complexity region is highlighted in pink. (**B**) Predicted secondary structure of *AfBBX*: α-helices (red), extended chains (blue), and random coils (black). (**C**) Predicted tertiary structure and sequence conservation analysis of AfBBX. The upper panel shows the 3D cartoon model of AfBBX generated by SWISS-MODEL. The lower panel presents the sequence alignment between AfBBX and the homologous protein D0EP05.1. The blue, green, and yellow background boxes collectively denote the predicted conserved B-box domains (typically involved in protein-protein interactions and zinc binding). Small arrows in the sequence alignment indicate the conserved zinc-coordinating residues (such as C, H, and D) characteristic of these B-box domains. The red background box indicates a putative nuclear localization signal (NLS), characterized by a cluster of basic amino acids.

Initially, a transcriptomic analysis was performed on *A. fruticosa* under stress conditions to identify differentially expressed genes. This analysis identified only one BBX family member, which showed significant stress-responsive upregulation and was therefore designated as *AfBBX.* The sequencing data are available at Bigsub database (https://bigd.big.ac.cn/gsub/) (accessed on 25 September 2024) with accession number CRA002113. The full-length coding sequence (CDS) of *AfBBX* was obtained from transcriptome sequencing data of *A. fruticosa* and subsequently cloned using RT-PCR. The complete CDS (1116 bp, encoding 371 amino acids) is provided as Supplementary File S1. SMART (http://smart.embl-heidelberg.de/) (accessed on 25 September 2024) analysis confirmed the presence of two conserved ZnF-BBOX domains (Figure 1A), a characteristic feature of BBX proteins. Secondary structure prediction using SWISS-MODEL (https://swissmodel.expasy.org/) (accessed on 25 September 2024) revealed that the AfBBX protein consists of four α-helix regions, seven extended-chain regions, and thirteen randomly coiled regions (Figure 1B). In addition, tertiary structure modeling demonstrated that the BBX domains form conserved zinc-binding folds (Figure 1C).

### 2.2. Organ-Specific and Stress-Responsive Expression of AfBBX

**Figure 2:**
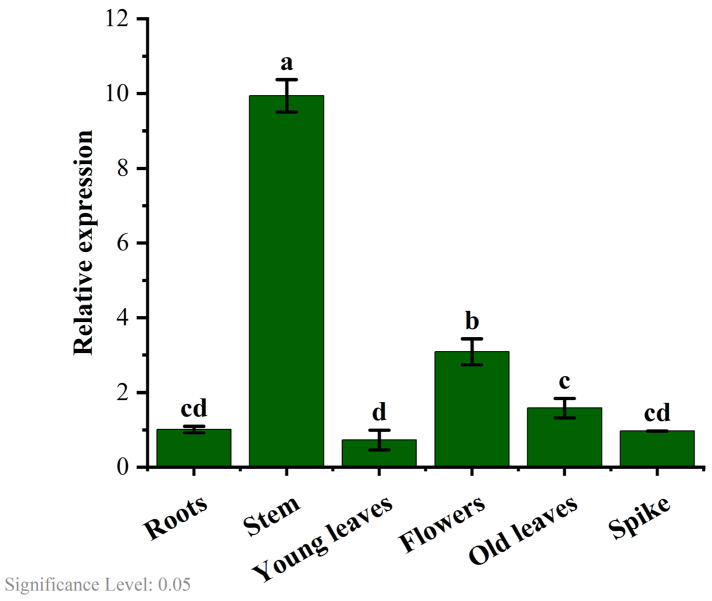
Organ-specific expression of *AfBBX* in *A. fruticosa*. qRT-PCR, with three biological replicates, was performed, following the extraction of total RNA from roots, stems, young leaves, old leaves, flowers, and spikes. Error bars represent standard errors. Lowercase letters (a, b, c, and d) indicate statistically significant differences (*p* < 0.05) determined by one-way ANOVA with Tukey’s HSD test.

According to an analysis of the organ-specific expression of *AfBBX* in *A. fruticosa* by qRT-PCR, *AfBBX* was expressed in all tested organs (roots, stems, young leaves, old leaves, flowers, and spikes). *AfBBX* exhibited the highest expression in stems, followed by flowers, old leaves, roots, and spikes. Expression in young leaves was lower than in old leaves, though this difference was less pronounced than that observed for roots and spikes (Figure 2). Overall, transcript levels were highest in stems and lowest in young leaves (Figure 2). Considering this organ-specific expression pattern, *AfBBX* might play distinct roles in different organs of *A. fruticosa*.

**Figure 3:**
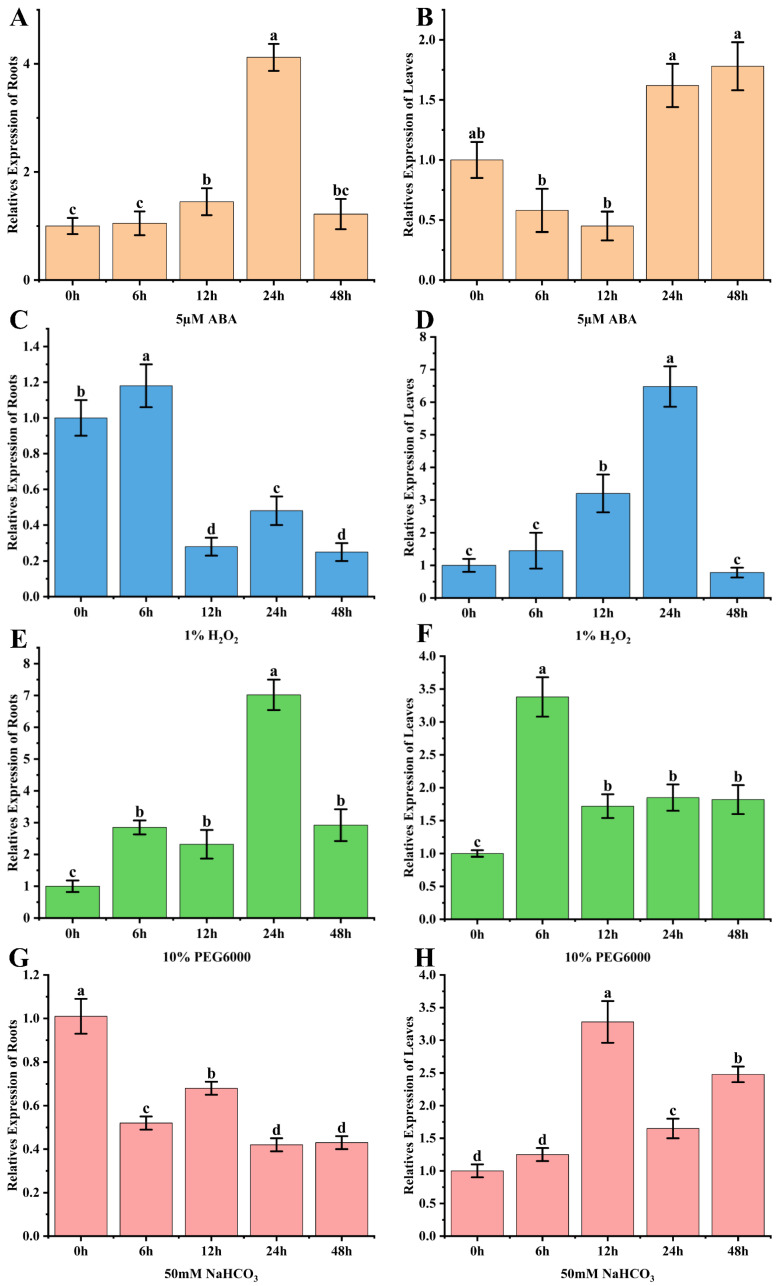
Expression patterns of *AfBBX* in roots and leaves of *A. fruticosa* under different stress treatments. (**A**,**B**) ABA (5 μM) treatment; (**C**,**D**) H_2_O_2_ (1%) treatment; (**E**,**F**) PEG600 (10%) treatment; (**G**,**H**) NaHCO_3_ (50 mM) treatment. qRT-PCR was performed with three biological replicates. Error bars represent standard errors. Lowercase letters (a, b, c, and d) indicate statistically significant differences (*p* < 0.05) determined by one-way ANOVA with Tukey’s HSD test.

To investigate the stress responsiveness of *AfBBX*, *A. fruticosa* seedlings were subjected to different treatments based on the experimental protocol, including 5 μM ABA (drought mimic), 1% H_2_O_2_ (oxidative stress), 10% PEG600 (osmotic stress), and 50 mM NaHCO_3_ (alkaline stress). *AfBBX* expression in roots and leaves was analyzed at 0, 6, 12, 24, and 48 h post-treatment (Figure 3).

Under ABA treatment, in roots, *AfBBX* expression gradually increased, peaking at 24 h, and then stabilized. In leaves, *AfBBX* expression first decreased at 6 h, followed by a gradual increase, with significantly higher levels at 24 h and 48 h compared to those at 0 h (Figure 3A,B). Under H_2_O_2_ treatment, *AfBBX* expression in roots exhibited significant decrease at 12 h and remained stable thereafter; in contrast, in leaves, its expression initially increased, peaked at 24 h, and then decreased (Figure 3C,D). Under 10% PEG600 treatment, *AfBBX* expression in roots was significantly upregulated, reaching a peak at 24 h, and remained higher than the control at 48 h; however, in leaves, *AfBBX* expression increased at 6 h, then decreased and stabilized (Figure 3E,F). Under NaHCO_3_ treatment, *AfBBX* expression in roots showed a clear reduction and stabilization after 24 h; in contrast, its expression in leaves was upregulated, peaking at 12 h and then decreasing (Figure 3G,H). Collectively, *AfBBX* might be dynamically regulated by various abiotic stresses, suggesting its involvement in multiple stress response pathways.

#### Subcellular Localization of AfBBX

**Figure 4:**
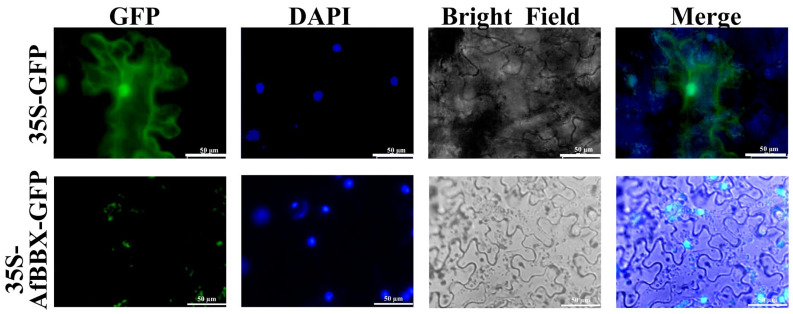
Subcellular localization of the AfBBX protein. Scale bars: 50 μm.

Given that TFs typically exert their functions in the nucleus, in our study, the subcellular localization of *AfBBX* was analyzed using a GFP fusion protein. Following transient expression of 35S-*AfBBX*-GFP in plant cells, it was observed with an exclusive localization of the GFP fluorescence (corresponding to *AfBBX*) to regions stained by DAPI (a nuclear marker; Figure 4). The nuclear localization of *AfBBX* was further confirmed by merged images of GFP, DAPI, and bright-field channels. These findings are consistent with AfBBX functioning as a transcription factor.

To investigate the subcellular distribution of *AfBBX* (a key step in unraveling its regulatory role as a TF), in our experiment, a 35S-*AfBBX*-GFP fusion construct was generated and transiently expressed in plant cells. Under the confocal microscope, it was found that:

- In the GFP channel, green fluorescence (indicating *AfBBX*-GFP) was concentrated in discrete, punctate structures (Figure 4, top and bottom panels, GFP column).
- In the DAPI channel, these structures colocalized with DAPI-stained nuclei (blue fluorescence; Figure 4, DAPI column).
- Merged images (Merge column) showed overlapping green (*AfBBX*-GFP) and blue (DAPI) signals, confirming the nuclear localization of *AfBBX*.

### 2.3. Enhanced Tolerance to Stress at the Germination and Seedling Stages by Overexpressing AfBBX

To confirm the successful generation of transgenic tobacco lines overexpressing AfBBX, we performed both molecular and antibiotic resistance assays. First, PCR analysis using gene-specific primers yielded the expected amplicon of approximately 1000 bp in the two independent transgenic lines (#1 and #2), but not in the wild-type negative control (Supplementary File S2 Figure S2). Subsequently, hygromycin resistance screening further validated the transgenic status of these lines (Supplementary File S2 Figure S3). Quantitative real-time PCR analysis confirmed that AfBBX was highly expressed in the transgenic lines (#1 and #2) compared to wild-type (WT) plants (Supplementary File S2 Figure S1). Two independent T3 transgenic lines (#1, #2) with high AfBBX expression levels were selected for subsequent experiments.

To investigate the function of *AfBBX* in stress tolerance, two independent T_3_ transgenic lines (#1, #2) with high *AfBBX* expression levels were selected for subsequent experiments.

#### Tolerance at the Germination Stage

**Figure 5:**
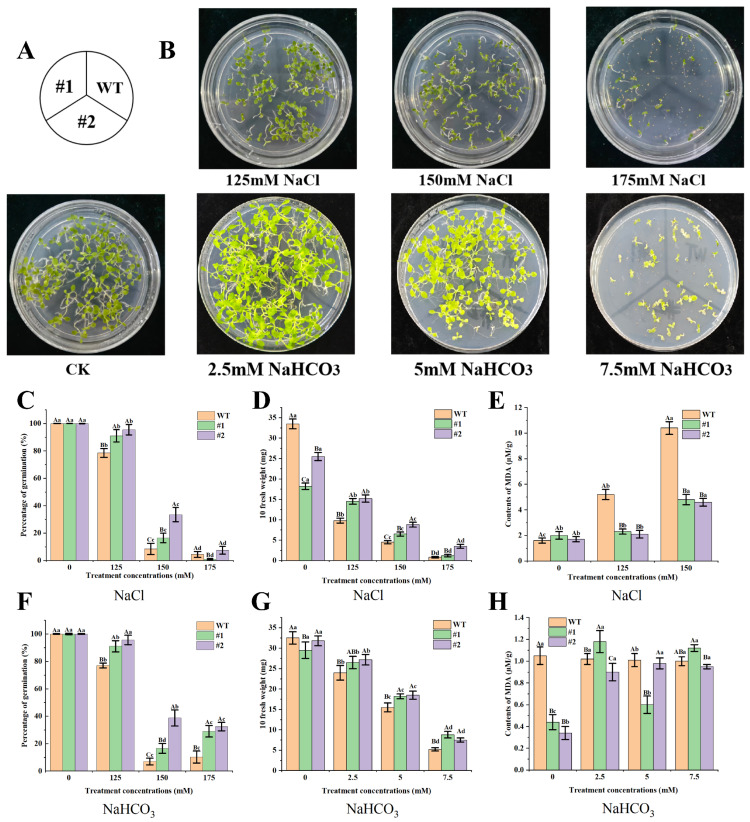
Physiological responses of WT and *AfBBX*-overexpressing transgenic tobacco to NaCl and NaHCO_3_ stress. (**A**) Schematic diagram illustrating the arrangement of WT and transgenic seeds (#1 and #2) in the Petri dishes. (**B**) Representative photographs of seedling phenotypes under control conditions (CK) and various concentrations of NaCl (125, 150, 175 mM) and NaHCO_3_ (2.5, 5.0, 7.5 mM). (**C**) Germination rates of WT and transgenic lines under 0–175 mM NaCl stress. (**D**) Fresh weights (×10 mg) of seedlings under NaCl stress. (**E**) MDA content (μM/g FW) in seedlings under NaCl stress. (**F**) Germination rates of WT and transgenic lines under 0–7.5mM NaHCO_3_ stress. (**G**) Fresh weights (×10 mg) of seedlings under NaHCO_3_ stress. (**H**) MDA content (μM/g FW) in seedlings under NaHCO_3_ stress. CK: Well-watered Control, 0 mM NaCl, 0 mM NaHCO_3_. Error bars represent standard errors of three biological replicates. Lowercase letters (a, b, c, d) indicate statistically significant differences (*p* < 0.05) among different concentrations within the same genotype, while uppercase letters (A, B, C, D) indicate statistically significant differences among different genotypes at the same concentration, as determined by two-way ANOVA with Tukey’s HSD test.

The germination rates of WT and transgenic tobacco seeds were evaluated under NaCl, and NaHCO_3_ treatments (Figure 5). Under normal conditions (0 mM stress), germination rates showed no significant difference between WT and transgenic lines. Under NaCl treatments (125, 150, and 175 mM), transgenic lines exhibited higher germination rates than WT (Figure 5C,D). Similarly, transgenic tobacco showed enhanced germination performance under NaHCO_3_ treatments (2.5, 5, and 7.5 mM; Figure 5E,F). Thus, *AfBBX* overexpression improved tolerance to salt and alkaline stress during germination.

To further characterize stress tolerance in *AfBBX*-overexpressing tobacco, germination rates, seedling fresh weights, and MDA content were measured under NaCl and NaHCO_3_ stresses (Figure 5). Under NaCl stress (0–175 mM), germination rates of WT decreased sharply with increasing salt concentrations. Specifically, at 150 mM NaCl, WT germination dropped to ~5%, whereas transgenic lines #1 and #2 maintained ~20% and ~30%, respectively (Figure 5A). Correspondingly, seedling fresh weights of transgenic lines were significantly higher than WT at 125–175 mM NaCl. At 175 mM NaCl, fresh weight of line #2 was approximately five-fold higher than that of WT (Figure 5B,C), confirming improved growth under salt stress. Under NaHCO_3_ stress (0–7.5 mM), WT germination was severely inhibited (~5% at 7.5 mM), whereas transgenic lines #1 and #2 retained germination rates of ~20% and ~30%, respectively (Figure 5D). Seedling fresh weights of transgenic lines were also significantly greater than WT at 2.5–7.5 mM NaHCO_3_ (Figure 5E). Consistently, MDA content (an indicator of membrane damage) was significantly lower in transgenic lines compared to WT under NaHCO_3_ stress. Specifically, at 5 mM NaHCO_3_, MDA levels in line #1 were approximately 40% of those in WT (Figure 5F). Collectively, improved germination, enhanced growth, and reduced membrane lipid peroxidation indicate that *AfBBX* overexpression enhances tolerance to both salt and alkaline stress in tobacco.

### 2.4. Growth and Physiological Adaptation of Transgenic Tobacco to Abiotic Stress

**Figure 6:**
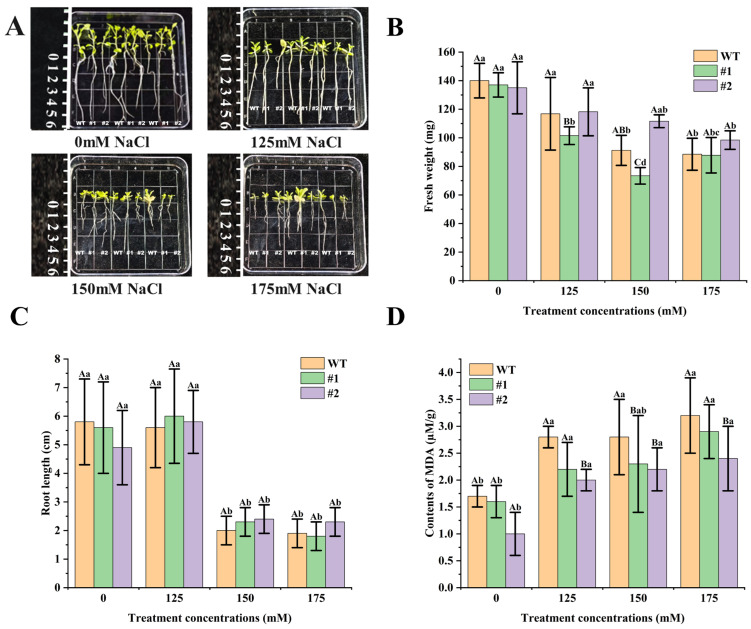
Growth traits of WT and *AfBBX*-overexpressing transgenic tobacco under NaCl stress. (**A**) Phenotypes of WT and transgenic lines (#1, and #2) after 15 days of cultivation in 1/2 MS medium supplemented with 0, 125, 150, and 175 mM NaCl treatments. Scale bar = 1 cm. (**B**) Seedling fresh weight. (**C**) Seedling root length. Error bars represent standard errors of three biological replicates. (**D**) MDA content (μM/g FW) in seedlings. Error bars represent standard errors of three biological replicates. Lowercase letters (a, b, c, d) indicate statistically significant differences (*p* < 0.05) among different concentrations within the same genotype, while uppercase letters (A, B, C) indicate statistically significant differences among different genotypes at the same concentration, as determined by two-way ANOVA with Tukey’s HSD test.

WT and transgenic lines (#1, and #2) were grown for 15 days in 1/2 MS medium containing 0, 125, 150, or 175 mM NaCl for subsequent assessment of the salt tolerance of *AfBBX* transgenic tobacco at the seedling stage. Under non-stress conditions (0 mM NaCl), WT and transgenic lines showed no significant differences in growth (phenotype, fresh weight, and root length) (Figure 6A–C). When exposed to NaCl stress, WT seedlings exhibited severely inhibited growth, manifesting as sharp decrease in the root length and fresh weight with increasing NaCl concentration, with evident leaf chlorosis (Figure 6A). Conversely, transgenic lines retained more vigorous growth, as supported by significantly longer root lengths of transgenic lines (Figure 6C), and higher fresh weight (Figure 6B) than WT at 125–150 mM NaCl. Even at 175 mM NaCl, transgenic lines maintained greater fresh weights and root lengths than WT.

Subsequently, according to the measurement of MDA content, under NaCl stress, there were markedly increased MDA levels in WT, while transgenic lines showed significantly lower MDA content (Figure 6D). Altogether, overexpressing *AfBBX* could enhance tobacco tolerance to NaCl stress by mitigating growth inhibition and reducing membrane damage.

**Figure 7:**
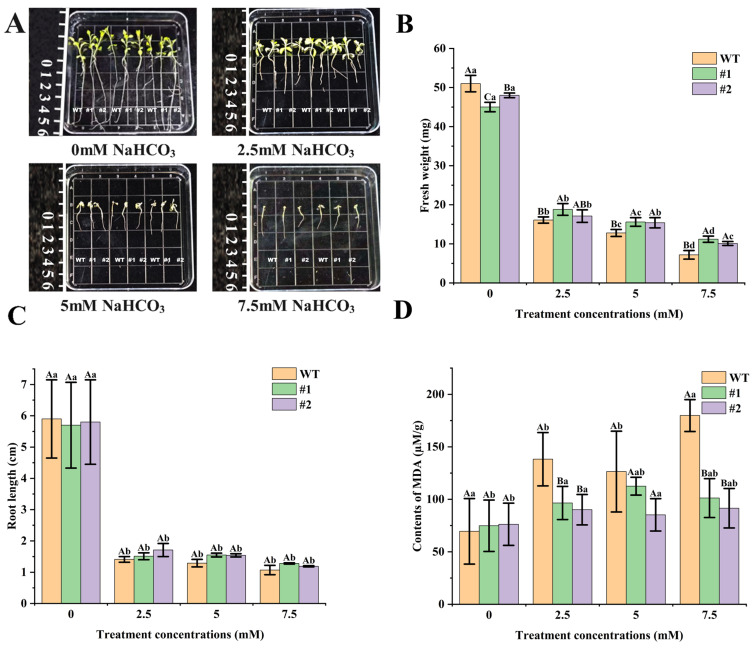
Growth and physiological responses of WT and *AfBBX*-overexpressing transgenic tobacco under NaHCO_3_ stress. (**A**) Phenotypes of WT and transgenic lines (#1, and #2) after 15 days of cultivation in 1/2 MS medium supplemented with 0, 2.5, 5, and 7.5 mM NaHCO_3_. Scale bar = 1 cm. (**B**) Seedling fresh weight (mg/plant). (**C**) Seedling root length (cm). (**D**) MDA content (μM/g FW) in seedlings. Error bars represent standard errors of three biological replicates. Lowercase letters (a, b, c, d) indicate statistically significant differences (*p* < 0.05) among different concentrations within the same genotype, while uppercase letters (A, B, C) indicate statistically significant differences among different genotypes at the same concentration, as determined by two-way ANOVA with Tukey’s HSD test.

The 1/2 MS medium with 0, 2.5, 5, or 7.5 mM NaHCO_3_ was used for 15 days of WT and *AfBBX* transgenic tobacco cultivation to evaluate alkaline tolerance. In the absence of stress (0 mM NaHCO_3_), WT and transgenic lines showed similar growth (Figure 7A–C). Under NaHCO_3_ stress, both seedlings presented with suppressed growth, but with less severe effects in transgenic lines, as supported by significantly longer root lengths and greater fresh weights than WT at 2.5–5 mM NaHCO_3_ (Figure 7B,C). Even at 7.5 mM NaHCO_3_, transgenic lines retained higher fresh weights than WT (Figure 7B).

MDA content analysis showed that NaHCO_3_ stress induced a greater increase in MDA levels in WT compared to transgenic lines (Figure 7D), indicating that AfBBX overexpression alleviated membrane lipid peroxidation under alkaline stress. Consequently, *AfBBX* improved tobacco tolerance to NaHCO_3_ stress by maintaining growth and reducing oxidative damage.

**Figure 8:**
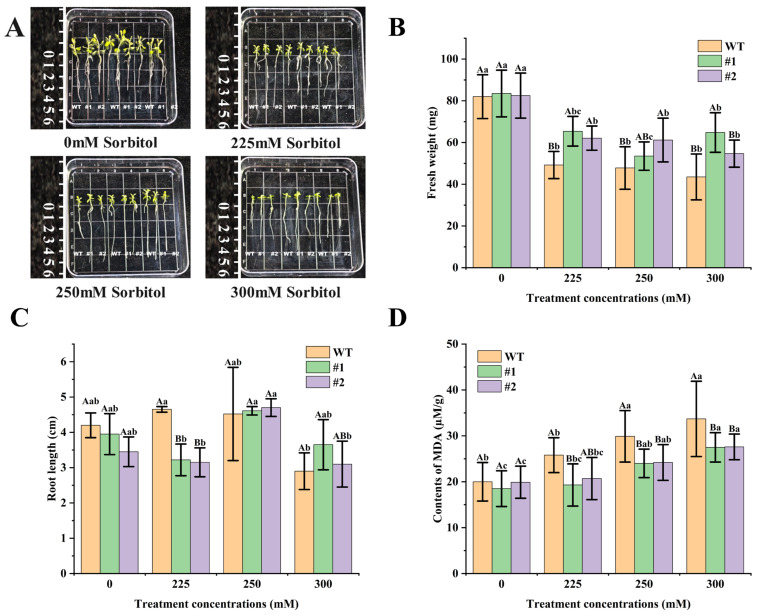
Growth and physiological responses of WT and *AfBBX*-overexpressing transgenic tobacco under sorbitol stress. (**A**) Phenotypes of WT and transgenic lines (#1, and #2) after 15 days of cultivation in 1/2 MS medium supplemented with 0, 225, 250, and 300 mM sorbitol. Scale bar = 1 cm. (**B**) Seedling fresh weight (mg/plant). (**C**) Seedling root length (cm). (**D**) MDA content (μM/g FW) in seedlings. Error bars represent standard errors of three biological replicates. Lowercase letters (a, b, c) indicate statistically significant differences (*p* < 0.05) among different concentrations within the same genotype, while uppercase letters (A, B) indicate statistically significant differences among different genotypes at the same concentration, as determined by two-way ANOVA with Tukey’s HSD test.

To assess osmotic tolerance, WT and AfBBX transgenic tobacco seedlings were cultured for 15 days on 1/2 MS medium containing 0, 225, 250, or 300 mM sorbitol. Sorbitol was selected instead of PEG, a common osmotic agent, as PEG can interfere with agar solidification, whereas sorbitol ensures consistent gel formation. Under non-stress conditions (0 mM sorbitol), WT and transgenic lines showed comparable growth parameters (Figure 8A–C). However, exposure to sorbitol stress severely inhibited WT growth, resulting in reduced root lengths, lower fresh weights, and apparent leaf wilting with increasing sorbitol concentrations (Figure 8A). In contrast, transgenic lines exhibited healthier growth, showing significantly greater root lengths and higher fresh weights than WT at 225–300 mM sorbitol (Figure 8B,C).

Furthermore, sorbitol stress caused a more substantial increase in MDA levels in WT than in transgenic lines (Figure 8D), indicating reduced membrane damage in *AfBBX*-overexpressing plants. Thus, *AfBBX* overexpression enhanced tobacco tolerance to drought-like stress by preserving growth and mitigating oxidative damage.

### 2.5. Improved Drought and Abiotic Tolerance in Mature Tobacco by Overexpressing AfBBX

**Figure 9:**
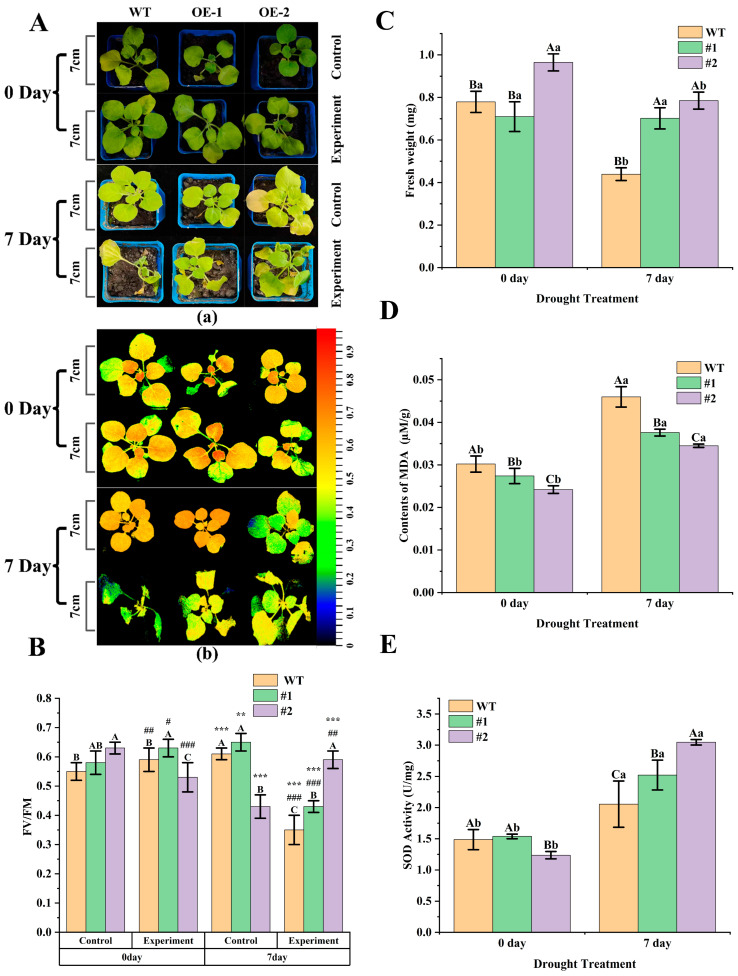
Phenotypic and physiological responses of WT and *AfBBX*-overexpressing transgenic tobacco under drought stress. (**A**) (**a**) Phenotypes of WT and transgenic lines (#1, and #2) at 0 and 7 days of natural drought treatment; (**b**) corresponding chlorophyll fluorescence (Fv/Fm) imaging on the left (scale bar: 7 cm), with a color gradient on the right indicating Fv/Fm values (0–1). (**B**) Fv/Fm (photosystem II efficiency) of seedlings at 0 and 7 days of drought stress. (**C**) Seedling fresh weight. (**D**) MDA content (μM/g FW). (**E**) SOD activity (U/mg prot). Error bars represent standard errors of three biological replicates. Asterisks (*) indicate statistically significant differences between day 7 and day 0 when other levels are the same (** *p* < 0.01, *** *p* < 0.001); hash symbols (#) indicate statistically significant differences between control and experimental groups when other levels are the same (# *p* < 0.05, ## *p* < 0.01, ### *p* < 0.001). Lowercase letters (a, b) indicate statistically significant differences (*p* < 0.05) among different times within the same genotype, while uppercase letters (A, B, C) indicate statistically significant differences among different genotypes at the same time, as determined by two-way ANOVA (**C**–**E**) or three-way ANOVA (**B**) with Tukey’s HSD test.

To evaluate osmotic tolerance at the mature seedling stage, WT and *AfBBX* transgenic tobacco were subjected to natural drought for 7 days (Figure 9). Under non-stress conditions (0 day), there existed no significant differences in phenotype, Fv/Fm, fresh weight, MDA content, or SOD activity between WT and transgenic lines (Figure 9A–E). Following 7 days of drought, in contrast to severe wilting and chlorosis in WT seedlings, transgenic lines retained relatively turgid leaves [Figure 9A(a)]. Chlorophyll fluorescence imaging confirmed a sharper decline in Fv/Fm (photosynthetic damage) in WT than in transgenic lines [Figure 9A(b),B].

Quantitatively, transgenic lines maintained significantly higher fresh weight (Figure 9C) and SOD activity (Figure 9E), but less MDA (Figure 9D), than WT after drought treatment. It could be speculated that *AfBBX* overexpression could enhance osmotic tolerance, which might be related to the preservation of the photosynthetic efficiency, reduction in the membrane lipid peroxidation, and strengthening of the antioxidant capacity.

**Figure 10:**
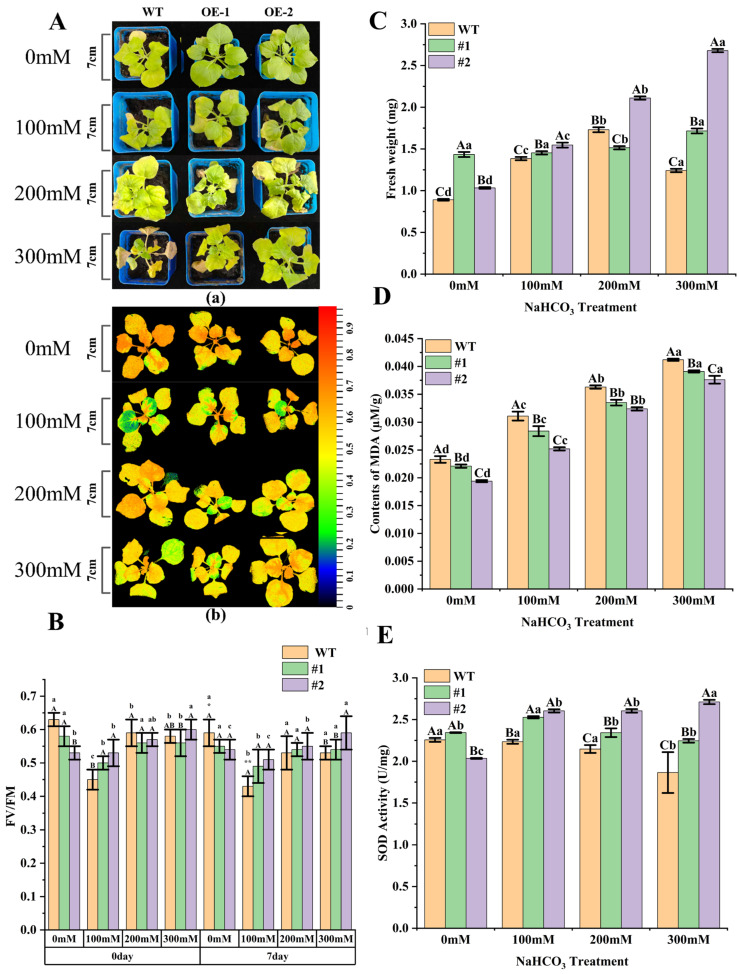
Phenotypic and physiological responses of WT and *AfBBX*-overexpressing transgenic tobacco under NaHCO_3_ stress. (**A**) (**a**) Phenotypes of WT and transgenic lines (#1, and #2) after 7 days of 0, 100, 200, 300 mM NaHCO_3_ treatment; (b) corresponding chlorophyll fluorescence (Fv/Fm) imaging on the left (scale bar: 7 cm), with color gradient on the right indicating Fv/Fm values (0–1). (**B**) Fv/Fm of seedlings at 0 and 7 days of NaHCO_3_ treatment. (**C**) Seedling fresh weight. (**D**) MDA content (μM/g FW). (**E**) SOD activity (U/mg prot). Error bars represent the standard error from three biological replicates. Asterisks (*) indicate statistically significant differences between day 7 and day 0 when other levels are the same (* *p* < 0.05, ** *p* < 0.01,), lowercase letters (a, b, c, d) indicate significant differences (*p* < 0.05) among different time points within the same genotype. Uppercase letters (A, B, C) indicate significant differences among genotypes at the same time (two-way ANOVA for (**C**–**E**); three-way ANOVA for (**B**), with Tukey’s HSD test).

To assess alkaline tolerance, WT and *AfBBX* transgenic tobacco were provided with 7 days of treatment using 0–300 mM NaHCO_3_ (Figure 10). Under non-stress conditions (0 mM NaHCO_3_), there were no statistical differences in the growth and physiological parameters of WT and transgenic lines (Figure 10A–E). In the context of increased NaHCO_3_ concentration, WT seedlings exhibited progressive wilting and chlorosis, while transgenic lines retained healthier morphology [Figure 10A(a)]. Further, chlorophyll fluorescence imaging showed that WT revealed remarkably reduced Fv/Fm compared to transgenic lines [Figure 10A(b),B]. Conversely, transgenic lines maintained higher fresh weight (Figure 10C) and SOD activity (Figure 10E), as well as lower MDA content (Figure 10D) than WT at 100–300 mM NaHCO_3_. Even under severe alkaline stress at 300 mM NaHCO_3_, transgenic lines exhibited less growth inhibition and oxidative damage than WT. Hence, NaHCO_3_ stress tolerance might be enhanced by overexpressing *AfBBX* through the protection of the photosynthetic system and mitigation of oxidative damage.

**Figure 11:**
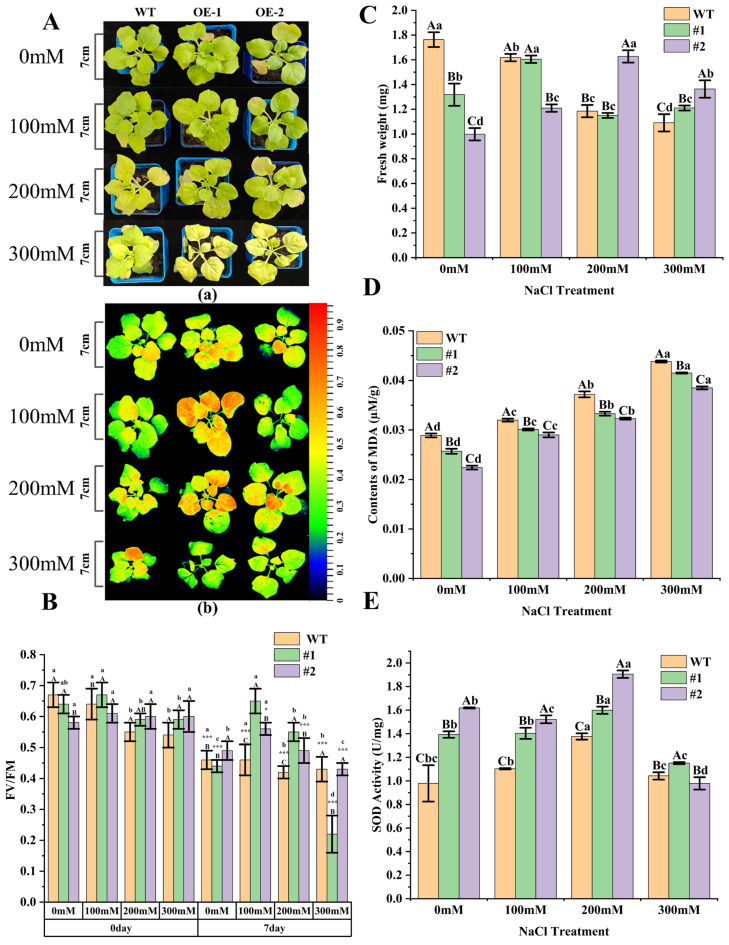
Phenotypic and physiological responses of WT and *AfBBX*-overexpressing transgenic tobacco under NaCl stress. (**A**) (**a**) Phenotypes of WT and transgenic lines (#1, and #2) after 7 days of 0, 100, 200, 300 mM NaCl treatment; (**b**) corresponding chlorophyll fluorescence (Fv/Fm) imaging on the left (scale bar: 7 cm), with color gradient on the right indicating Fv/Fm values (0–1). (**B**) Fv/Fm of seedlings at 0 and 7 days of NaCl treatment. (**C**) Seedling fresh weight. (**D**) MDA content (μM/g FW). (**E**) SOD activity (U/mg prot). Error bars represent standard errors of three biological replicates. Asterisks (*) indicate statistically significant differences between day 7 and day 0 when other levels are the same (* *p* < 0.05, *** *p* < 0.001); Lowercase letters (a, b, c, d) indicate statistically significant differences (*p* < 0.05) among different times within the same genotype, while uppercase letters (A, B, C) indicate statistically significant differences among different genotypes at the same time, as determined by two-way ANOVA (**C**–**E**) or three-way ANOVA (**B**) with Tukey’s HSD test.

To evaluate salt tolerance, WT and *AfBBX* transgenic tobacco were treated with 0–300 mM NaCl for 7 days (Figure 11). At 0 mM NaCl, WT and transgenic lines showed no significant differences in growth or physiology (Figure 11A–E). WT seedlings exhibited severe wilting and chlorosis, while transgenic lines retained more vigorous growth as NaCl concentration increased (Figure 11A(a)). Meanwhile, chlorophyll fluorescence imaging revealed that WT displayed a sharper decline in Fv/Fm compared to transgenic lines (Figure 11A(b),B). Notably, at 300 mM NaCl, both transgenic lines exhibited a marked decline in Fv/Fm compared to WT, which may be attributed to a metabolic burden imposed by constitutive AfBBX overexpression, potentially diverting energy from photosystem repair under extreme stress [23].

Transgenic lines maintained higher fresh weight (Figure 11C) and SOD activity (Figure 11E), as well as lower MDA content (Figure 11D), than WT at 100–300 mM NaCl. Under severe salt stress at 300 mM NaCl, transgenic lines showed less growth retardation and oxidative damage than WT. Taken together, overexpressing *AfBBX* could boost NaCl stress tolerance by preserving photosynthetic efficiency, reducing membrane lipid peroxidation, and strengthening antioxidant defense.

## 3. Discussion

Drought and salt–alkali stresses significantly limit plant growth and productivity, highlighting the importance of identifying stress-resistant genes for molecular breeding [24]. BBX proteins are recognized as key regulators of plant stress responses, with corresponding functional characterizations reported in various plant species [6,7]. In this study, we cloned the *AfBBX* gene from *A. fruticosa*, a stress-tolerant woody plant, and provided preliminary evidence that heterologous expression of *AfBBX* is associated with enhanced drought and salt–alkali stress tolerance in transgenic tobacco.

Bioinformatics analysis indicated that *AfBBX* contains conserved ZnF-BBOX domains essential for BBX protein function [6]. The analysis also revealed that *AfBBX* is evolutionarily conserved with BBX proteins from other plants, suggesting similar biological functions. Based on the transcriptome sequencing data of *A. fruticosa*, only one *BBX* family member was identified under current assembly and annotation conditions. However, plant *BBX* gene families typically contain multiple members. For example, the reported number of *BBX* members in soybean varies considerably due to differences in genome assembly quality, annotation stringency, and structural domain inclusion criteria [25]. Therefore, we cannot definitively conclude that *AfBBX* is the sole *BBX* family member in *A. fruticosa*, as additional homologs might exist that were not captured by transcriptome-based identification. Tissue-specific expression analysis revealed high *AfBBX* expression in *A. fruticosa* stems. Although this observation is correlative, it aligns with the known roles of stems in water transport and mechanical support during stress exposure. Stems are critical for long-distance water transport under drought, and elevated expression of stress-related genes in stems may help to maintain xylem hydraulic conductivity and reduce cavitation [26]. Therefore, this finding should be interpreted as preliminary, warranting further functional validation, such as tissue-specific promoter activity assays or stem-targeted gene-silencing experiments. Under various stresses, *AfBBX* expression was dynamically regulated in roots and leaves, indicating its potential role in the perception and response to multiple abiotic stresses. The functional diversity of BBX proteins has been further underscored by genome-wide identification in species such as ginger [27] and peanut [28], where specific BBX members are implicated in responses to ABA, drought, heat, and salt stresses. Previous studies [8,9] documented similar expression patterns for other *BBX* genes, such as *AtBBX24* in *Arabidopsis* and *OsBBX11* in rice, which respond to salt and drought stresses.

Furthermore, our subcellular localisation analysis revealed that *AfBBX*-GFP fusion protein is predominantly localised in the nucleus, although a weaker cytoplasmic signal is also consistently observed. This nuclear-enriched distribution pattern is typical for many *BBX* transcription factors, which exert their regulatory functions by modulating gene expression in the nucleus [7,29]. The additional cytoplasmic signal, which is notably weaker than that of free GFP, may reflect either a genuine but minor cytoplasmic pool or passive diffusion due to the relatively small size of the AfBBX protein. Similar nucleo-cytoplasmic distributions have been reported for other BBX proteins, such as AtBBX24 and AtBBX18, and are thought to contribute to the fine-tuning of stress responses by regulating the availability of these transcription factors in the nucleus [30,31].

Transgenic tobacco overexpressing *AfBBX* exhibited enhanced tolerance to drought and salt–alkali stresses at germination and seedling stages. Typically, plant stress tolerance can be primarily evaluated by germination rate, root length, and fresh weight [32]. Higher germination rates and improved growth performance of transgenic lines under stress indicate that *AfBBX* promotes seed germination and seedling growth in adverse environments. At maturity, transgenic tobacco maintained higher photosynthetic efficiency (Fv/Fm) under drought and salt–alkali stresses, suggesting *AfBBX* protects the photosynthetic system from stress-induced damage. Photosynthesis is highly sensitive to abiotic stress; thus, preserving photosynthetic capacity is crucial for plant survival [33]. While the consistent phenotype observed in the two independent lines provides a strong indication of *AfBBX* function, we recognize that analysis of additional independent transgenic events is warranted to definitively rule out positional effects. Future work will focus on characterizing a broader population of transgenic lines alongside molecular dissection of the underlying regulatory pathways.

ROS accumulation commonly occurs in plants under abiotic stress, causing oxidative damage to proteins, lipids, and nucleic acids [34]. Plants respond by activating antioxidant defense systems, including enzymes such as SOD and POD [35]. In this study, transgenic tobacco overexpressing *AfBBX* showed increased SOD and POD activities and reduced MDA content under stress conditions. This suggests that *AfBBX* enhances ROS scavenging and decreases membrane lipid peroxidation. Similarly, other stress-responsive transcription factors, such as *PaBBX* [14] and light-responsive transcription factors like *SlBBX20*, enhance stress tolerance by regulating antioxidant enzyme activities and maintaining photosynthetic capacity [36]. Therefore, *AfBBX* likely improves stress tolerance by modulating the antioxidant defense system.

## 4. Materials and Methods

### 4.1. Plant Materials and Growth Conditions

Seeds of *A. fruticosa* were harvested from the East Road Green Belt in Ningjiang District, Songyuan City, Jilin Province, China. Tobacco seeds were preserved by the Key Laboratory of Saline Alkali Land Vegetation Restoration and Reconstruction, Ministry of Education, Northeast Forestry University. For each treatment and time point, five independent seedlings (*n* = 5) were sampled for expression analysis and physiological measurements. For processing, seeds were sown in a mixture of vermiculite and perlite (1:2, *v*/*v*) after surface sterilization with 75% ethanol for 30 s, followed by 0.1% HgCl_2_ for 5 min, and rinsed with sterile water. Seedlings were grown in a growth chamber at 25 °C with a 16 h light/8 h dark photoperiod and 70% relative humidity.

Meanwhile, tobacco (*Nicotiana tabacum* cv. Xanthi) was used for genetic transformation. Tobacco seeds were grown on 1/2 MS medium after sterilization, and seedlings were grown in the same growth chamber as *A. fruticosa*.

### 4.2. Stress Treatments for Expression Analysis

The following stress treatments were employed on four-week-old *A. fruticosa* seedlings:

- Drought stress: irrigation with 5 μM ABA solution.
- Oxidative stress: irrigation with 1% H_2_O_2_ solution.
- Osmotic stress: irrigation with 10% PEG solution.
- Alkaline stress: irrigation with 50 mM NaHCO_3_ solution.

Roots and leaves were sampled at 0, 6, 12, 24, and 48 h post-treatment, followed by immediate freezing in liquid nitrogen and storage at −80 °C for RNA extraction.

### 4.3. Cloning of AfBBX

Total RNA was extracted from *A. fruticosa* leaves using TRIzol reagent (Invitrogen, Carlsbad, CA, USA) according to the manufacturer’s instructions. First-strand cDNA synthesis was carried out using the PrimeScript RT Reagent Kit with gDNA Eraser (TaKaRa, Dalian, China). The full-length coding sequence (CDS) of AfBBX was obtained from transcriptome sequencing data of *A. fruticosa* and provided as Supplementary File S1. The full-length *AfBBX* CDS was amplified using specific primers (*AfBBX*-F: 5′-ATGGCGGAGAGCGGCGAG-3′; *AfBBX*-R: 5′-TCAGTTGGTGGTGGTGGTG-3′) designed based on transcriptome data. The PCR product was then cloned into the pMD18-T vector (TaKaRa) and sequenced by Sangon Biotech (Shanghai, China).

### 4.4. Bioinformatics Analysis

The amino acid sequence of *AfBBX* was subjected to analysis of physical and chemical properties using ExPASy (https://web.expasy.org/protparam/) (accessed on 25 September 2024). The prediction of conserved domains was performed using SMART (http://smart.embl-heidelberg.de/) (accessed on 25 September 2024), and the prediction of secondary and tertiary structures was carried out using SWISS-MODEL (https://swissmodel.expasy.org/ (accessed on 25 September 2024)).

### 4.5. Quantitative Real-Time Polymerase Chain Reaction (qRT-PCR)

qRT-PCR was performed using the SYBR Premix Ex Taq II Kit (TaKaRa) on an ABI 7500 Real-Time PCR System (Applied Biosystems, Foster City, CA, USA). The *A. fruticosa* Tubulin gene (AfTubu) (AfTubu-F: 5′-ACAAGGCGGTTAAGGTTGGT-3′; AfTubu-R: 5′-GTTCTGGGCTTGGTTCCCTT-3′) was used as an internal reference. The 2^−ΔΔCt^ method was employed to quantify the relative expression levels of *AfBBX* [37]. Each sample was analyzed with three biological replicates and three technical replicates.

### 4.6. Construction of Plant Expression Vector and Genetic Transformation

The full-length CDS of *AfBBX* was inserted into the pBI121 vector under the control of the CaMV 35S promoter. The resulting recombinant vector (pBI121-*AfBBX*) was transformed into *Agrobacterium tumefaciens* strain EHA105 using the freeze–thaw method. Tobacco transformation was performed using the leaf disc method [38]. Transgenic tobacco plants were selected on kanamycin (50 mg/L)-containing 1/2 MS medium. T_3_ generation seeds were used for subsequent experiments.

### 4.7. Stress Tolerance Assays

#### 4.7.1. Germination Assay

Wild-type (WT) and transgenic tobacco seeds were sterilized and sown on 1/2 MS medium supplemented with NaCl (0, 125, 150, and 175 mM) and NaHCO_3_ (0, 2.5, 5, and 7.5 mM) at different concentrations. Seeds were incubated in a growth chamber at 25 °C with a 16 h light/8 h dark photoperiod. Germination was defined as the emergence of the radicle through the seed coat. The rate of germination was recorded daily for 10 days.

#### 4.7.2. Seedling Assay

WT and transgenic tobacco seeds were grown for 7 days after sowing on 1/2 MS medium. Seedlings with uniform growth were transferred to 1/2 MS medium supplemented with sorbitol (0, 225, 250, and 300 mM), NaCl (0, 125, 150, and 175 mM) and NaHCO_3_ (0, 2.5, 5, and 7.5 mM). Root length and fresh weight were measured after 15 days of growth. Leaves were sampled for the measurement of superoxide dismutase (SOD) activity and malondialdehyde (MDA) content using commercial kits (Nanjing Jiancheng Bioengineering Institute, Nanjing, China) according to the manufacturer’s instructions.

#### 4.7.3. Mature Plant Stress Assay

WT and transgenic tobacco seedlings were grown for an additional 4 weeks after transplanting into pots filled with a mixture of soil and vermiculite (2:1, *v*/*v*). In the assessment under different stresses, watering was specifically withheld for 7 days to induce drought stress [39]. Meanwhile, for salt and alkaline stresses, plants were respectively irrigated with NaCl solution (100, 200, and 300 mM), and NaHCO_3_ solution (100, 200, and 300 mM) every 2 days for 7 days. After recording phenotypes, chlorophyll fluorescence parameters (Fv/Fm) were measured using a FluorCam open chlorophyll fluorescence imaging system (PSI, Brno, Czech Republic). In addition, leaves were sampled to measure SOD activity and MDA content using commercial kits (Nanjing Jiancheng Bioengineering Institute, Nanjing, China) according to the manufacturer’s instructions.

### 4.8. Statistical Analysis

All experiments were performed with three biological replicates. Data were analyzed using SPSS 25.0 (IBM, Armonk, NY, USA). Depending on the experimental design, one-way, two-way, or three-way ANOVA was conducted, followed by Tukey’s HSD test for multiple comparisons (*p* < 0.05). One-way ANOVA was used to assess differences among multiple groups under a single factor; two-way ANOVA was applied to evaluate the effects of two independent factors (e.g., genotype and treatment concentration) and their interaction; three-way ANOVA was used for factorial designs involving three factors (e.g., genotype, treatment, and time point), followed by simple effects analysis to examine specific interactions. All error bars in the figures represent standard deviation (SD). Significant differences between WT and transgenic lines were determined accordingly.

## 5. Conclusions

This study focused on cloning and characterizing the *AfBBX* gene from *A. fruticosa*, demonstrating its function in enhancing drought and salt–alkali stress tolerance in transgenic tobacco. *AfBBX* expression is dynamically regulated under various abiotic stresses and is highly expressed in stems of *A. fruticosa*. Overexpression of AfBBX improved tobacco germination rates, root lengths, and fresh weights under stress conditions. Transgenic tobacco plants also maintained higher photosynthetic efficiency, increased antioxidant enzyme activity, and lower membrane lipid peroxidation under drought and salt–alkali stress. Thus, *AfBBX* enhances stress tolerance by protecting the photosynthetic system and strengthening antioxidant defenses. This study identifies AfBBX as a promising candidate gene for stress-resistant plant breeding, provides new insights into the molecular mechanisms underlying BBX-mediated stress resistance, and offers valuable genetic resources for developing stress-tolerant crops and forest trees. Future research should identify downstream target genes of *AfBBX* and explore its interactions with other proteins, thereby elucidating the regulatory networks involving *AfBBX* in plant stress responses.

## Data Availability

Data will be made available on request.

## Supplementary Materials

The following supporting information can be downloaded at https://www.mdpi.com/article/10.3390/ijms27114902/s1.

## Abbreviations

The following abbreviations are used in this manuscript:

GFP	Green fluorescent protein Autofluorescent protein widely used as a reporter in molecular and cellular biology.
MDA	Malondialdehyde Marker of lipid peroxidation and oxidative stress.
SOD	Superoxide dismutase Antioxidant enzyme catalyzing the dismutation of superoxide radicals.

## References

[1] Pan Y., Birdsey R.A., Fang J., Houghton R., Kauppi P.E., Kurz W.A., Phillips O.L., Shvidenko A., Lewis S.L., Canadell J.G. (2011). A large and persistent carbon sink in the world’s forests. Science.

[2] Pennisi E. (2022). Global drought experiment reveals the toll on plant growth. Science.

[3] Allen C.D., Macalady A.K., Chenchouni H., Bachelet D., McDowell N., Vennetier M., Kitzberger T., Rigling A., Breshears D.D., Hogg E.H. (2010). A global overview of drought and heat-induced tree mortality reveals emerging climate change risks for forests. For. Ecol. Manag..

[4] Anderegg W.R.L., Trugman A.T., Badgley G., Anderson C.M., Bartuska A., Ciais P., Cullenward D., Field C.B., Freeman J., Goetz S.J. (2020). Climate-driven risks to the climate mitigation potential of forests. Science.

[5] Zhang H., Zhu J., Gong Z., Zhu J.K. (2022). Abiotic stress responses in plants. Nat. Rev. Genet..

[6] Yamaguchi-Shinozaki K., Shinozaki K. (2006). Transcriptional regulatory networks in cellular responses and tolerance to dehydration and cold stresses. Annu. Rev. Plant Biol..

[7] Gangappa S.N., Botto J.F. (2014). The BBX family of plant transcription factors. Trends Plant Sci..

[8] Yadav A., Ravindran N., Singh D., Rahul P.V., Datta S. (2020). Role of Arabidopsis BBX proteins in light signaling. J. Plant Biochem. Biotechnol..

[9] Heng Y., Lin F., Jiang Y., Ding M., Yan T., Lan H., Zhou H., Zhao X., Xu D., Deng X.W. (2019). B-Box Containing Proteins BBX30 and BBX31, Acting Downstream of HY5, Negatively Regulate Photomorphogenesis in Arabidopsis. Plant Physiol..

[10] Jiang L., Wang Y., Li Q.F., Björn L.O., He J.X., Li S.S. (2012). Arabidopsis STO/BBX24 negatively regulates UV-B signaling by interacting with COP1 and repressing HY5 transcriptional activity. Cell Res..

[11] Shen T., Xu F., Chen D., Yan R., Wang Q., Li K., Zhang G., Ni L., Jiang M. (2024). A B-box transcription factor OsBBX17 regulates saline-alkaline tolerance through the MAPK cascade pathway in rice. New Phytol..

[12] An J.P., Wang X.F., Zhang X.W., Bi S.Q., You C.X., Hao Y.J. (2020). Apple B-box protein BBX37 regulates jasmonic acid mediated cold tolerance through the JAZ-BBX37-ICE1-CBF pathway and undergoes MIEL1-mediated ubiquitination and degradation. New Phytol..

[13] Li J., Ai G., Wang Y., Ding Y., Hu X., Liang Y., Yan Q., Wu K., Huang R., Chen C. (2024). A truncated B-box zinc finger transcription factor confers drought sensitivity in modern cultivated tomatoes. Nat. Commun..

[14] Yang H., Mao X., Xu P., Fan G. (2025). Characterization, stress responses and protein-protein interactions of the Paulownia BBX transcription factor. Ind. Crops Prod..

[15] Huang S., Chen C., Xu M., Wang G., Xu L.A., Wu Y. (2021). Overexpression of Ginkgo BBX25 enhances salt tolerance in transgenic Populus. Plant Physiol. Biochem..

[16] Deng H., Zhang Y., Manzoor M.A., Sabir I.A., Han B., Song C. (2024). Genome-scale identification, expression and evolution analysis of B-box members in Dendrobium huoshanense. Heliyon.

[17] Gong Z. (2021). Plant abiotic stress: New insights into the factors that activate and modulate plant responses. J. Integr. Plant Biol..

[18] Jiang H., Chen X., Xu G., Chen J., Song D., Lv M., Guo H., Chen J. (2025). Plant Adaptation and Soil Shear Strength: Unraveling the Drought Legacy in *Amorpha fruticosa*. Plants.

[19] Cao Q., Li J., Xiao H., Cao Y., Xin Z., Yang B., Liu T., Yuan M. (2020). Sap flow of *Amorpha fruticosa*: Implications of water use strategy in a semiarid system with secondary salinization. Sci. Rep..

[20] Chen L., Wang R., Hu X., Wang D., Wang Y., Xue R., Wu M., Li H. (2024). Overexpression of wheat C2H2 zinc finger protein transcription factor TaZAT8-5B enhances drought tolerance and root growth in Arabidopsis thaliana. Planta.

[21] Liu T., Wang Y., Li X., Che H., Zhang Y. (2024). LpNAC5 positively regulates drought, salt and alkalinity tolerance of Lilium pumilum. Gene.

[22] Li S., Khoso M.A., Wu J., Yu B., Wagan S., Liu L. (2024). Exploring the mechanisms of WRKY transcription factors and regulated pathways in response to abiotic stress. Plant Stress.

[23] Dougherty L., Cooper B., Bunce J., Vinyard B., Stommel J. (2025). Biomass and yield in *Solanum lycopersicum* expressing a synthetic photorespiration pathway. J. Am. Soc. Hortic. Sci..

[24] Xu C.Y., Liu H.Y., Ciais P., Hartmann H., Camarero J.J., Wu X.C., Hammond W.M., Allen C.D., Chen F.H. (2024). Enhanced Drought Exposure Increasingly Threatens More Forests Than Observed. Earths Future.

[25] Shan B.H., Bao G.H., Shi T.R., Zhai L.L., Bian S.M., Li X.Y. (2022). Genome-wide identification of BBX gene family and their expression patterns under salt stress in soybean. BMC Genom..

[26] Chaves M.M., Flexas J., Pinheiro C. (2009). Photosynthesis under drought and salt stress: Regulation mechanisms from whole plant to cell. Ann. Bot..

[27] Shi J.Y., Tang Y.R., Li H.L., Xing H.T. (2025). The BBX Family and Their Response to Abiotic Stress in Ginger (*Zingiber officinale* Roscoe). BMC Genom..

[28] Tang H., Yuan C., Shi H., Liu F., Shan S., Wang Z., Sun Q., Sun J. (2024). Genome-Wide Identification of Peanut B-Boxs and Functional Characterization of AhBBX6 in Salt and Drought Stresses. Plants.

[29] Xu D., Jiang Y., Li J., Lin F., Holm M., Deng X.W. (2016). BBX21, an Arabidopsis B-box protein, directly activates HY5 and is targeted by COP1 for 26S proteasome-mediated degradation. Proc. Natl. Acad. Sci. USA.

[30] Chu Z., Wang X., Li Y., Yu H., Li J., Lu Y., Li H., Ouyang B. (2016). Genomic Organization, Phylogenetic and Expression Analysis of the B-BOX Gene Family in Tomato. Front. Plant Sci..

[31] Cheng X., Lei S., Li J., Tian B., Li C., Cao J., Lu J., Ma C., Chang C., Zhang H. (2024). In Silico Analysis of the Wheat BBX Gene Family and Identification of Candidate Genes for Seed Dormancy and Germination. BMC Plant Biol..

[32] Yu Z., Yan H., Liang L., Zhang Y., Yang H., Li W., Choi J., Huang J., Deng S. (2021). A C2H2-Type Zinc-Finger Protein from Millettia pinnata, MpZFP1, Enhances Salt Tolerance in Transgenic Arabidopsis. Int. J. Mol. Sci..

[33] Baker N.R. (2008). Chlorophyll fluorescence: A probe of photosynthesis in vivo. Annu. Rev. Plant Biol..

[34] Nadarajah K.K. (2020). ROS Homeostasis in Abiotic Stress Tolerance in Plants. Int. J. Mol. Sci..

[35] Hasanuzzaman M., Bhuyan M.H.M.B., Anee T.I., Parvin K., Nahar K., Mahmud J.A., Fujita M. (2019). Regulation of Ascorbate-Glutathione Pathway in Mitigating Oxidative Damage in Plants under Abiotic Stress. Antioxidants.

[36] Ma Y., Xu Z., Wang L., Ding R., Zhang Y., Wang J., Wang P., Yao W., Li X., Li G. (2024). The light-responsive transcription factor SlBBX20 improves saline-alkali resistance of Solanum lycopersicum by affecting photosynthetic capacity, antioxidant capacity, and osmotic adjustment. Environ. Exp. Bot..

[37] Livak K.J., Schmittgen T.D. (2001). Analysis of relative gene expression data using real-time quantitative PCR and the 2^−ΔΔCt^ method. Methods.

[38] Horsch R.B., Fry J.E., Hoffmann N.L., Eichholtz D., Rogers S.G., Fraley R.T. (1985). A simple and general method for transferring genes into plants. Science.

[39] Guo J., Zhao Y., Cheng H., Yu R., Gu B., Wang Q., Zhang J., Li S., Guan Q. (2024). Enhancing Plant Stress Tolerance: The Role of LcWRKY40 Gene in Drought and Alkaline Salt Resistance in Tobacco and Yeast. Int. J. Mol. Sci..

