# Supplementary material for: Functional Characterization of AfBBX from Amorpha fruticosa in Enhancing Osmotic and Salt–Alkali Tolerance in Transgenic Tobacco
Source: Int J Mol Sci. 2026 May 28;27(11):4902. doi: 10.3390/ijms27114902 (PMC13256605; doi:10.3390/ijms27114902)
Supplement: Supplementary file 1 [file ijms-27-04902-s001.zip › Supplementary/Supplementary1.pptx]

## Slide 1
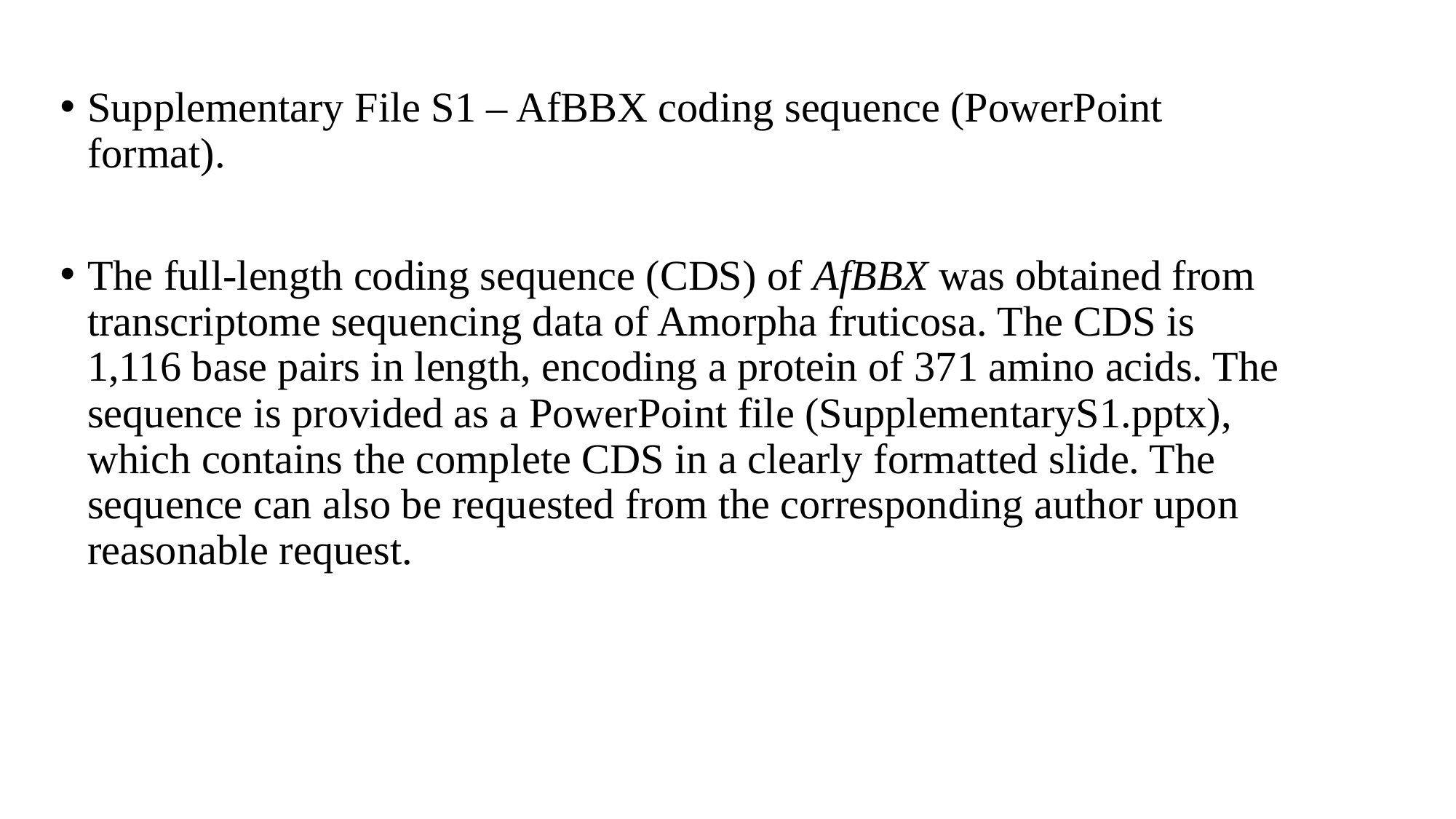

Supplementary File S1 – AfBBX coding sequence (PowerPoint format).
The full-length coding sequence (CDS) of AfBBX was obtained from transcriptome sequencing data of Amorpha fruticosa. The CDS is 1,116 base pairs in length, encoding a protein of 371 amino acids. The sequence is provided as a PowerPoint file (SupplementaryS1.pptx), which contains the complete CDS in a clearly formatted slide. The sequence can also be requested from the corresponding author upon reasonable request.

## Slide 2
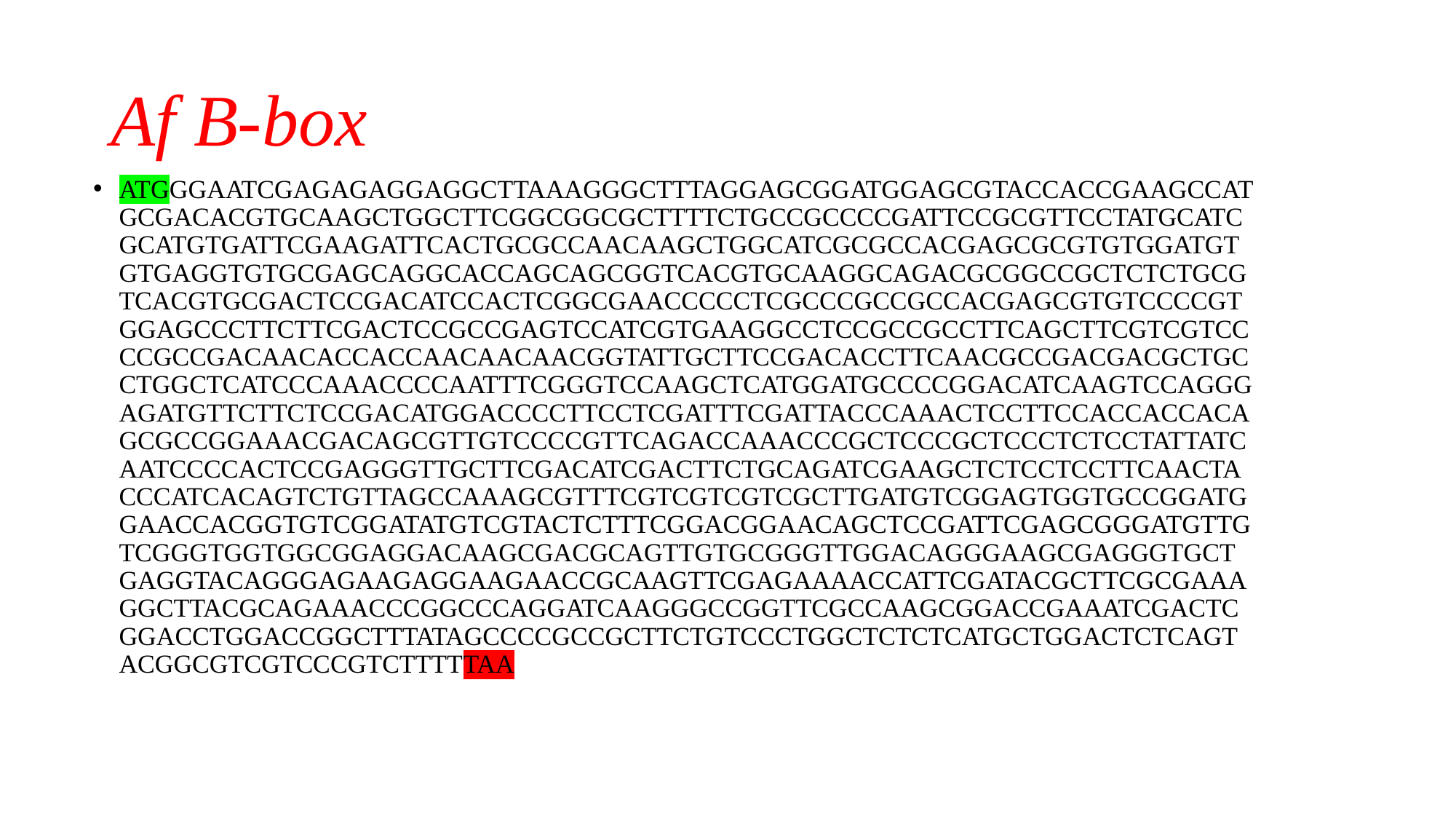

# Af B-box
ATGGGAATCGAGAGAGGAGGCTTAAAGGGCTTTAGGAGCGGATGGAGCGTACCACCGAAGCCATGCGACACGTGCAAGCTGGCTTCGGCGGCGCTTTTCTGCCGCCCCGATTCCGCGTTCCTATGCATCGCATGTGATTCGAAGATTCACTGCGCCAACAAGCTGGCATCGCGCCACGAGCGCGTGTGGATGTGTGAGGTGTGCGAGCAGGCACCAGCAGCGGTCACGTGCAAGGCAGACGCGGCCGCTCTCTGCGTCACGTGCGACTCCGACATCCACTCGGCGAACCCCCTCGCCCGCCGCCACGAGCGTGTCCCCGTGGAGCCCTTCTTCGACTCCGCCGAGTCCATCGTGAAGGCCTCCGCCGCCTTCAGCTTCGTCGTCCCCGCCGACAACACCACCAACAACAACGGTATTGCTTCCGACACCTTCAACGCCGACGACGCTGCCTGGCTCATCCCAAACCCCAATTTCGGGTCCAAGCTCATGGATGCCCCGGACATCAAGTCCAGGGAGATGTTCTTCTCCGACATGGACCCCTTCCTCGATTTCGATTACCCAAACTCCTTCCACCACCACAGCGCCGGAAACGACAGCGTTGTCCCCGTTCAGACCAAACCCGCTCCCGCTCCCTCTCCTATTATCAATCCCCACTCCGAGGGTTGCTTCGACATCGACTTCTGCAGATCGAAGCTCTCCTCCTTCAACTACCCATCACAGTCTGTTAGCCAAAGCGTTTCGTCGTCGTCGCTTGATGTCGGAGTGGTGCCGGATGGAACCACGGTGTCGGATATGTCGTACTCTTTCGGACGGAACAGCTCCGATTCGAGCGGGATGTTGTCGGGTGGTGGCGGAGGACAAGCGACGCAGTTGTGCGGGTTGGACAGGGAAGCGAGGGTGCTGAGGTACAGGGAGAAGAGGAAGAACCGCAAGTTCGAGAAAACCATTCGATACGCTTCGCGAAAGGCTTACGCAGAAACCCGGCCCAGGATCAAGGGCCGGTTCGCCAAGCGGACCGAAATCGACTCGGACCTGGACCGGCTTTATAGCCCCGCCGCTTCTGTCCCTGGCTCTCTCATGCTGGACTCTCAGTACGGCGTCGTCCCGTCTTTTTAA
