# Supplementary material for: Functional Characterization of AfBBX from Amorpha fruticosa in Enhancing Osmotic and Salt–Alkali Tolerance in Transgenic Tobacco
Source: Int J Mol Sci. 2026 May 28;27(11):4902. doi: 10.3390/ijms27114902 (PMC13256605; doi:10.3390/ijms27114902)
Supplement: Supplementary file 1 [file ijms-27-04902-s001.zip › Supplementary/Supplementary2.pdf]

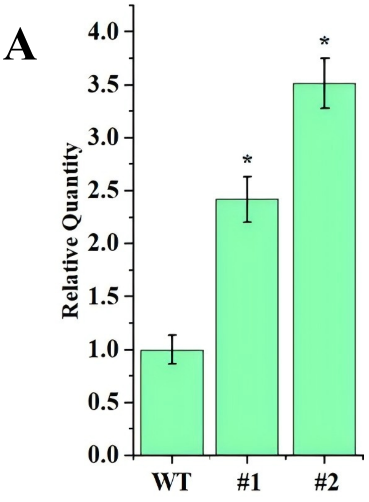

Supplementary2 Figure S1:Expression levels of *AfBBX* in wild-type (WT) and transgenic tobacco lines. qRT-PCR was performed with three biological replicates.

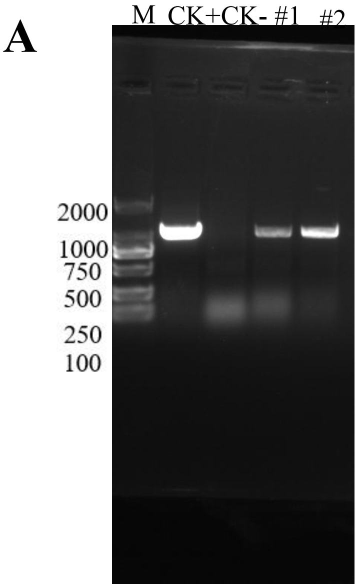

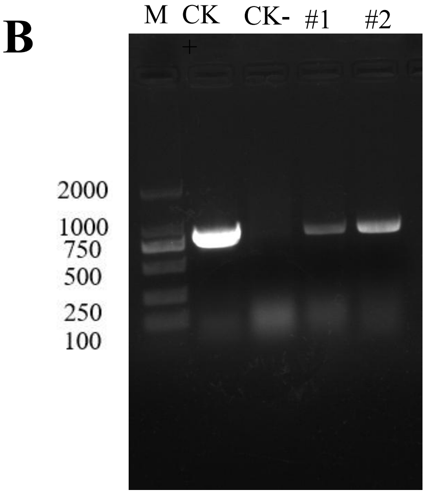

Supplementary Figure S2. PCR identification of transgenic Af B-box tobacco lines. (A) PCR amplification results using a 2000 bp DNA marker. Lanes from left to right: DNA marker (M), positive control (CK+), negative control (CK-), and two independent transgenic lines (#1 and #2). The results indicate that specific bands are present in both transgenic lines.

Supplementary Figure S3. Hygromycin resistance identification of transgenic Af B-box tobacco lines. (A) PCR amplification results using a 2000 bp DNA marker. Lanes from left to right: DNA marker (M), positive control (CK+), negative control (CK-), and two independent transgenic lines (#1 and #2). The results indicate that a specific band of the expected size (approximately 1000 bp) is present in both transgenic lines.
